# Persistent Atraumatic Knee Pain in a Teenage Female with Bony Protuberance Secondary to Hook-Shaped Osteochrondroma

**DOI:** 10.1155/2021/3088992

**Published:** 2021-12-18

**Authors:** Adityanarayan Rao, Joshua Pryor, Jaclyn Otero, Molly Posa

**Affiliations:** ^1^University of Florida College of Medicine, Gainesville, FL 32610, USA; ^2^University of Florida-Pediatrics Department, Gainesville, FL 32610, USA

## Abstract

A 13-year-old female presented at her pediatrician's office with a complaint of sharp, intermittent, right-sided knee pain that had been present for the previous three days without any known trauma and no association with activity. Her medical history was significant for fractures, and on physical exam, there was a hard mass palpated on the medial aspect of her distal thigh that was nontender, nonmobile, and without overlying skin changes. The plain radiograph findings were consistent with a hook-shaped osteochondroma of the right medial distal metaphysis. Orthopedics recommended conservative management with continued ibuprofen for pain and six-week follow-up with repeat radiograph to evaluate for progression. The follow-up radiograph showed no interval growth. However, due to continued pain, the patient had surgical excision of the osteochondroma six months after initial presentation, allowing her to finish her current soccer season. The surgery was successful, and the patient did well after operation with no residual pain.

## 1. Background

Osteochondromas are the most common type of benign bone lesions accounting for nearly 30% of benign bone tumors. This condition is more common in males than in females at an approximate ratio of 2 : 1 and most frequently presents in children under 21 years of age [1]. This case highlights the need for holistic evaluation of each patient and under what circumstances to proceed with surgical intervention for this condition.

## 2. Case Presentation

A 13-year-old female presents at her pediatrician's office with a complaint of sharp, intermittent, right-sided knee pain that has been present for the previous three days. The patient first noticed the pain when she awoke three days ago. She denies any recent physical activity or known trauma. The pain occurs 2-3 times a day and is rapid in onset, lasting anywhere from 30 minutes to 3 hours. There is no association with time of day, activity, or rest. She states it feels like “someone hit her with a hammer,” and she has not found any aggravating factors. ibuprofen provides only minimal relief. The adolescent reports that she has recently had a growth spurt. The pain has not hindered her daily activities, and she has not noticed knee swelling, redness, or decreased range of motion. On pertinent review of systems, she denies fevers, chills, night sweats, weight loss, rash, palpitations, abdominal pain, vomiting, diarrhea, constipation, back pain, or pain in any additional joints.

Her medical history is significant for right radial and ulnar fractures requiring short arm casting after closed reduction, as well as a 5^th^ metatarsal fracture (Jones fracture) on her right foot. All of these occurred due to sports-related injuries and falls. She is not currently taking any medication other than ibuprofen as needed for knee pain. Her family history is negative for autoimmune disorders and childhood cancer.

**Figure 1:**
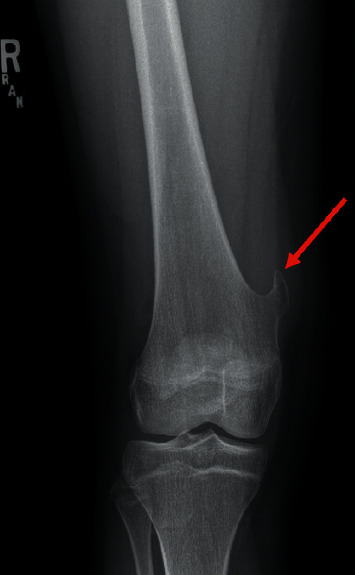
An anterior posterior radiograph of the patient's right knee with limited view of the femur showing the hook-shaped osteochondroma (red arrow).

**Figure 2:**
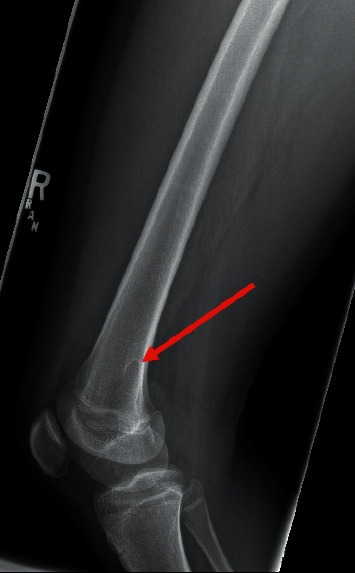
Lateral view of the right knee with the osteochondroma (red arrow).

On physical exam, there is a hard mass palpable on the medial aspect of her distal thigh that is nontender, nonmobile, and without overlying skin changes. She has full painless range of motion of her right knee on both extension and flexion, with negative anterior and posterior drawer, Lachman's, valgus and varus manipulation, and Thessaly's tests. There is no joint line, tibial plateau, patellar, or quadriceps tendon tenderness. There is no motor deficit, and there is normal sensation in the right lower extremity. The patient has normal gait. Due to a visual and palpable mass being present on physical examination, a radiograph of the right knee and femur is obtained with concerns for osteochondroma versus malignancy. The plain radiograph findings are consistent with a hook-shaped osteochondroma of the right medial distal metaphysis (Figures 1 and 2), and the patient was referred to orthopedics due to the significant pain.

## 3. Outcome

**Figure 3:**
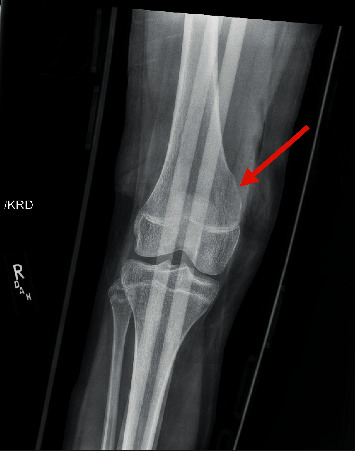
Plain radiograph of the right knee status after surgical excision of the osteochondroma with an immobilizer in place.

Orthopedics recommended conservative management with continued ibuprofen for pain and six-week follow-up with repeat radiograph to evaluate for progression. Physical therapy was not recommended during the six-week observation period. The patient continued to have pain consistent with initial presentation throughout the interceding six weeks. The follow-up radiograph showed no interval growth. Due to continued pain, the patient had surgical excision of the osteochondroma six months after initial presentation, allowing her to finish her current soccer season. The surgery was successful, and the patient did well after operation with no residual pain (Figure 3).

## 4. Discussion

Classic presentation of osteochondroma includes a single painless palpable mass located at the ends of the long bones or along the axial skeleton found incidentally on imaging after a trauma. The most common sites of growth are the ends of the long bones with special affinity for the distal and proximal femur as well as the proximal humerus [2]. While these lesions are usually painless, their frequency in the pediatric population and the possibility for pain and restriction of movement emphasize the importance of further clinical evaluation.

The lesion itself consists of a bony spur (sessile or pedunculated shape) with a cartilaginous cap, usually located at the epiphysis or metadiaphysis of the bone. The exact cause of formation is unknown, but osteochondroma pathophysiology manifests as a peripheral chondroblast growing outward from a metaphysis forming a cap over the bone [3]. The cartilaginous cap serves as the source of growth which typically continues until skeletal maturity occurs. Malignant changes are rare and occur in about 1% of the patient population: if this does occur, it typically occurs at 20–30 years of age with transformation of the lesion to chondrosarcoma [3, 4]. However, an autosomal dominant condition Multiple Hereditary Osteochondromatosis (germline mutations in EXT1 or EXT2 tumor suppressor genes) manifests as multiple lesions with a significantly higher probability of progression to malignancy (1% to 20%) [2, 5].

For a child presenting with a palpable bony mass, further investigation is recommended to rule out malignancy. The most common methodology of imaging is conventional radiology with specific attention to the anatomic location and the presence of transition zones [1]. A key radiological finding in osteochondroma is mineralization of the matrix, without which diagnosis is more difficult. In these cases, a computer tomography (CT) or magnetic resonance imaging (MRI) to identify additional features of osteochondromas, including endosteal scalloping, thick periosteal reaction, and cortical hook, is recommended [1].

Most cases of osteochondromas do not require surgical treatment, as osteochondromas will cease to grow after the patient reaches skeletal maturity. As such, first-line treatment involves conservative observation with imaging to monitor growth [6]. Surgical excisions are primarily reserved for patients with pain and/or reduced range of motion: persistent, significant pain was present in our patient, which is why surgical resection was recommended. In addition, cases of deformity and potential malignant transformation should be treated surgically [6].

Prognosis for osteochondromas, in both surgical resection as well as conservative management, is excellent. If surgical resection is considered, it is recommended after skeletal maturity occurs to decrease the risk of recurrence [4]. If surgery is necessary prior to skeletal maturity, due to pain or restriction of movement, a partial excision is recommended to preserve the physis, so as not to affect growth potential [7].

Our patient is an excellent reflection of the above findings. After diagnosis with an osteochondroma, the patient was managed conservatively for six months with the use of non-steroidal anti-inflammatory drugs before finally opting for surgical excision due to pain. Further follow-up demonstrated no residual pain and full range of motion reflecting the curative ability of surgical intervention for osteochondroma treatment.

## Data Availability

The research publications used to support the findings of this study are included within the article.

## References

[1] Gaumer G. R., Weinberg D. S., Collier C. D., Getty P. J., Liu R. W. (2017). An osteological study on the prevalence of osteochondromas. *The Iowa Orthopaedic Journal*.

[2] Hakim D. N., Pelly T., Kulendran M., Caris J. A. (2015). Benign tumours of the bone: a review. *Journal of Bone Oncology*.

[3] Copley L., Dormans J. P. (1996). Benign pediatric bone tumors. *Pediatric Clinics of North America*.

[4] Hameetman L., Bovée J. V., Taminiau A. H., Kroon H. M., Hogendoorn P. C. (2004). Multiple osteochondromas: clinicopathological and genetic spectrum and suggestions for clinical management. *Hereditary Cancer in Clinical Practice*.

[5] Baig M. N., O’Malley S., Fenelon C., Kaar K. (2019). Osteochondroma of acromioclavicular joint. *BMJ Case Reports*.

[6] Bovée J. V. (2008). Multiple osteochondromas. *Orphanet Journal of Rare Diseases*.

[7] Chin K. R., Kharrazi F. D., Miller B. S., Mankin H. J., Gebhardt M. C. (2000). Osteochondromas of the distal aspect of the tibia or fibula. *Journal of Bone and Joint Surgery American Volume*.

